# The Effectiveness of a Neurofeedback-Assisted Mindfulness Training Program Using a Mobile App on Stress Reduction in Employees: Randomized Controlled Trial

**DOI:** 10.2196/42851

**Published:** 2023-10-03

**Authors:** Beomjun Min, Heyeon Park, Johanna Inhyang Kim, Sungmin Lee, Soyoung Back, Eunhwa Lee, Sohee Oh, Je-Yeon Yun, Bung-Nyun Kim, Yonghoon Kim, JungHyun Hwang, Sanghyop Lee, Jeong-Hyun Kim

**Affiliations:** 1 Department of Public Health Medical Services Seoul National University Bundang Hospital Seongnam Republic of Korea; 2 Liberal Arts College Dongduk Women’s University Seoul Republic of Korea; 3 Department of Psychiatry Hanyang University Medical Center Seoul Republic of Korea; 4 Department of Psychiatry Seoul National University Bundang Hospital Seongnam Republic of Korea; 5 Department of Psychiatry Seoul National University Hospital Seoul Republic of Korea; 6 Medical Research Collaborating Center Seoul Metropolitan Government Seoul National University Boramae Medical Center Seoul Republic of Korea; 7 Yeongeon Student Support Center Seoul National University College of Medicine Seoul Republic of Korea; 8 Department of Psychiatry Seoul National University College of Medicine Seoul Republic of Korea; 9 OMNI C&S Inc Seoul Republic of Korea; 10 Department of Public Health Medical Services Seoul National University Bundang Hospital Seongnam-si Republic of Korea; 11 Institute of Human Behavioral Medicine Seoul National University Medical Research Center Seoul Republic of Korea

**Keywords:** mindfulness, neurofeedback, stress, resilience, mobile app, employee

## Abstract

**Background:**

Mindfulness-based training programs have consistently shown efficacy in stress reduction. However, questions regarding the optimal duration and most effective delivery methods remain.

**Objective:**

This research explores a 4-week neurofeedback-assisted mindfulness training for employees via a mobile app. The study’s core query is whether incorporating neurofeedback can amplify the benefits on stress reduction and related metrics compared with conventional mindfulness training.

**Methods:**

A total of 92 full-time employees were randomized into 3 groups: group 1 received mobile mindfulness training with neurofeedback assistance (n=29, mean age 39.72 years); group 2 received mobile mindfulness training without neurofeedback (n=32, mean age 37.66 years); and group 3 were given self-learning paper materials on stress management during their first visit (n=31, mean age 38.65 years). The primary outcomes were perceived stress and resilience scales. The secondary outcomes were mindfulness awareness, emotional labor, occupational stress, insomnia, and depression. Heart rate variability and electroencephalography were measured for physiological outcomes. These measurements were collected at 3 different times, namely, at baseline, immediately after training, and at a 4-week follow-up. The generalized estimating equation model was used for data analysis.

**Results:**

The 4-week program showed significant stress reduction (Wald *χ*_2_^2^=107.167, *P*<.001) and improvements in psychological indices including resilience, emotional labor, insomnia, and depression. A significant interaction was observed in resilience (time × group, Wald *χ*_4_^2^=10.846, *P*=.02). The post hoc analysis showed a statistically significant difference between groups 1 (least squares mean [LSM] 21.62, SE 0.55) and 3 (LSM 19.90, SE 0.61) at the posttraining assessment (*P*=.008). Group 1 showed a significant improvement (*P*<.001) at the posttraining assessment, with continued improvements through the 1-month follow-up assessment period (LSM 21.55, SE 0.61). Physiological indices were analyzed only for data of 67 participants (22 in group 1, 22 in group 2, and 23 in group 3) due to the data quality. The relaxation index (ratio of alpha to high beta power) from the right electroencephalography channel showed a significant interaction (time × group, Wald *χ*_2_^2^=6.947, *P*=.03), with group 1 revealing the highest improvement (LSM 0.43, SE 0.15) compared with groups 2 (LSM –0.11, SE 0.10) and 3 (LSM 0.12, SE 0.10) at the 1-month follow-up assessment.

**Conclusions:**

The study demonstrated that the neurofeedback-assisted group achieved superior outcomes in resilience and relaxation during the 4-week mobile mindfulness program. Further research with larger samples and long-term follow-up is warranted.

**Trial Registration:**

ClinicalTrials.gov NCT03787407; https://clinicaltrials.gov/ct2/show/NCT03787407

## Introduction

Mental stress is a major public health concern and has a high prevalence. Stress is suggested to negatively impact both the physical and mental health of individuals [1,2], ranging from cardiovascular mortality [3], musculoskeletal pain [4], type 2 diabetes [5], depression, and anxiety [6-8]. Work-related stress is associated with presenteeism, absenteeism, diminished productivity, and high employee turnovers [9-11]. According to an annual UK survey, stress, depression, or anxiety accounts for 44% of all work-related health issues, and 54% of all working days were lost due to a recent increase in poor health of employees [12]. Thus, the importance of stress management in employees can be seen at both the industrial and individual levels.

Recently, there has been an increase in the interest, and thereby increased research effort, surrounding the mindfulness-based training programs to reduce stress and improve mental wellness for employees [13]. Many organizations and corporations have adopted mindfulness-based programs to enhance their employees’ well-being and performance [14]. Several meta-analyses focusing on healthy individuals suggested that mindfulness-based training programs were able to reduce perceived stress, anxiety, and depression, as well as enhance the quality of life, spiritual values, and resilience [15,16]. Mindfulness-based training programs have also demonstrated benefits for clinicians, nursing staff, and health profession students who experience high stress levels and burnout rates [17-19]. The health benefits experienced by medical professionals could indirectly benefit patients by enhancing the quality of patient care and clinical practices [20]. However, despite these possible benefits, several issues, including training duration and delivery methods—and their varying effectiveness—have been addressed [17,21].

According to a qualitative study that reviewed 67 published studies [13], the most common method of delivery was in-person lectures (n=64, 96%), followed by audio recordings (n=39, 58%) and group discussions (n=34, 51%). Among these 67 studies, only 4 (6%) used online modules as a means of content delivery. However, with technological advancements and greater immersion of smartphones in daily life, there has recently been an increase in the interest in incorporating such technology into the field of mental health care [22]. With the advent of smartphone technology and mobile apps, health professionals are realizing their advantages and ways to enhance patient care. The advantages of mobile app–based approaches are increased accessibility, interactivity, and usability of various functions, such as treatment monitoring, appointment reminders, and recordkeeping [23,24]. These advantages could enhance patient’s or employees’ adherence to the mental health intervention program. A recent study suggested that mindfulness training using smartphone apps may provide immediate positive effects on mood and stress, as well as long-term benefits for attentional control [25].

In addition, in terms of training duration, a traditional 8-week mindfulness training program, such as mindfulness-based stress reduction (MBSR), may be a barrier to widespread organizational adoption and raises the question of whether a similar beneficial effect can be achieved with a shorter program [13]. A previous study reported that even a 4-day mindfulness training program was effective in reducing anxiety and fatigue, as well as increasing mindfulness [26]. In a systematic review, the correlation between the mean effect size and the number of in-class hours for MBSR training was not significant in clinical and nonclinical samples. This finding suggests that reduced—or shorter—class time may be worthwhile for those with time constraints or those with little to no motivation to participate [27].

Meanwhile, a recent meta-analysis concluded that the most consistent electroencephalography (EEG) findings associated with mindfulness training may be increased theta and alpha power [28]. Several previous studies have evaluated the relationship between mindfulness-based training and neurofeedback. Neurofeedback is a training method to control brain waves consciously, and an EEG is used to record these waves. During training, individuals are instructed to focus or relax, and EEG is presented as a type of visual stimuli in real time so that they can recognize their present state of brain activity. In a previous study, participants undergoing mindfulness meditation with alpha-neurofeedback demonstrated a higher alpha amplitude compared with their sham neurofeedback counterparts [29]. Other studies suggested that neurofeedback could mediate the effect of mindfulness meditation [30,31].

The purpose of this study is to verify the effects of a neurofeedback-assisted mindfulness training program delivered via a mobile app. We hypothesize that the neurofeedback-assisted mindfulness training might have augmenting effects on stress and related indices compared with mindfulness training alone.

## Methods

### Participants

Participants were recruited via advertisements at the Seoul National University Hospital and the Seoul National University Bundang Hospital between August 2018 and December 2018. The inclusion criteria were as follows: (1) age between 19 and 65 years; (2) total score of the Perceived Stress Scale (PSS) ≥14 at baseline, and (3) currently employed full-time. The exclusion criteria were (1) age <19 or >65 years; (2) having cognitive disorders, such as dementia or intellectual disability; (3) neurological disorders, such as epileptic disorders, stroke, brain tumors, or others; (4) current or previous history of psychosis, such as schizophrenia or bipolar I disorders; (5) current report of suicidal ideation; (6) other conditions that might influence the measurement of heart rate variability (HRV), such as cardiac or pulmonary disease; and (7) nonpharmacological psychiatric treatment, counseling, or meditation training within the past 6 months. As mental stress at the workplace is commonly associated with depression, anxiety, and insomnia [6-8], participants with these conditions, but in stable status, were not excluded, as long as the type and dosage of medication remained the same for the past 6 months. In the screening process, the Mini International Neuropsychiatric Interview (MINI) [32], which is a short, structured psychiatric interview designed to find a broad spectrum of psychiatric disorders as outlined in the Diagnostic and Statistical Manual of Mental Disorders, Fourth Edition, and International Classification of Diseases, Tenth Revision, was used to diagnose psychiatric disorders. The MINI was translated into Korean and has good validity and reliability [33]. The interviews were conducted by 2 psychologists with a master’s degree who were familiar with the tool. The estimated total sample size using G-Power was 90 (30 participants per group) with a predicted effect size of Cohen *d*=0.3, α level of .05, and a desired power of 0.7. Considering a dropout rate of 10%, we targeted to recruit 100 participants. Multimedia Appendix 1 presents the CONSORT (Consolidated Standards of Reporting Trials) checklist completed for this study.

### Assessments

Demographic information, including age, sex, marital status (unmarried or others), educational status (more than college education or less), and length of career (more than 3 years or less), was obtained using self-reported questionnaires.

As the primary psychological outcome measures, changes in the scores of the PSS and Brief Resilience Scale (BRS) were used. The secondary psychological outcomes were changes in the scores of scales assessing mindfulness (Korean version of the Mindfulness Attention Awareness Scale [K-MAAS]), emotional labor (Korean Emotional Labor Scale [KELS]), occupational stress (Korean Standard Occupational Stress Scale—short form [KOSS]), insomnia (Athens Insomnia Scale [AIS]), and depression (9-item Patient Health Questionnaire [PHQ-9]).

In its original form, as developed by Cohen et al [34], the PSS is a 14-item scale; however, a modified version with a 10-item scale is commonly used [35]. The questionnaire is rated on a 5-point Likert scale, with 0=never, 1=almost never, 2=sometimes, 3=fairly often, and 4=very often. Higher scores reflect higher levels of perceived stress.

The BRS, comprising 6 items and measured on a 5-point Likert Scale (1=strongly disagree to 5=strongly agree), was used to evaluate the resilience of each individual and assessed the ability to bounce back or recover from stress [36]. Higher scores indicated better resilience.

The K-MAAS was originally developed by Brown and Ryan [37] and validated by Kwon and Kim [38]. This 15-item scale focuses on the attention and awareness of mindful states, ranging from 1 (almost always) to 6 (almost never). Higher scores indicated higher levels of dispositional mindfulness.

The KELS was developed to assess the emotional labor of employees and was validated for its applicability to the Korean population by Lee et al [39]. Emotional labor was defined as the process by which employees have to control their feelings in accordance with the organizational demand and occupational role [40,41]. The KELS has 5 subscales: effort to control emotion (5 items), organizational monitoring system (4 items), demands of emotional labor (3 items), emotional damage (6 items), and organizational support system (7 items). Each item was rated on a 4-point Likert scale (1=not at all to 4=very much), with higher scores reflecting higher levels of emotional labor.

The KOSS-Short Form consists of 24 items, with each item rated on a 4-point Likert scale (1=never to 4=always). It was validated by Chang et al [42]. The scale is most commonly used and studied for the evaluation of job stress in South Korea. It consists of 7 subscales, including job demand (4 items), job control (4 items), interpersonal conflict (3 items), job insecurity (2 items), organizational system (4 items), lack of reward (3 items), and workplace environment (4 items). In this study, the sum of scores on each subscale was calculated and then converted to 100 points. Higher scores reflected higher levels of job stress.

Insomnia was assessed by the AIS, which consisted of 8 items with a 4-point Likert scale, with higher scores reflecting greater severity of insomnia [43].

The PHQ-9 was used to evaluate depressive symptoms, with higher scores indicating a higher level of depressive symptoms [44].

In addition, we measured the physiological variables, including HRV and EEG, which are widely used as stress-related physiological biosignals [45,46]. These physiological variables were acquired using headsets (neuroNicle FX2; Laxtha) [47]. In a previous work that used the FX2 device, the authors established the reliability of a prefrontal EEG marker from the device and reported that no significant differences in the mean values of the tested variables were observed between the prefrontal and occipital regions [48]. In our study, EEG and HRV were recorded simultaneously for 5 minutes for all participants in a resting state with eyes closed. Ocular, muscular, and other types of artifacts were detected by an automatic removal algorithm, and contaminated periods longer than 10 seconds were removed. For EEG, the band-pass filter (infinite impulse response Butterworth filters, high-pass filter: first order with fc=2.6 Hz; low-pass filter: eighth order with fc=43 Hz) with 3-43-Hz range was applied to the data. All data were digitized in the continuous recording mode (5 minutes of recording; 250-Hz sampling rate; 15-bit resolution). For HRV, the plethysmograph (PPG) waveform and the heartbeat time interval in milliseconds for each heartbeat were detected by the pulse wave sensor. These EEG and PPG raw data were transmitted to the host device in real time. In the host device, the fast Fourier transform was applied to calculate the power spectrum of the acquired EEG and HRV data. As a result, the power spectra of theta (4-8 Hz), alpha (8-12 Hz), low beta (12-15 Hz), beta (15-20 Hz), high beta (20-30 Hz), and gamma (30-40 Hz) frequency bands were calculated from the EEG data. In addition, the relaxation index (ratio of alpha to high beta power) and concentration index (ratio of low beta to theta power) were calculated from the EEG data. For HRV parameters, the power of low-frequency (LF; 0.04-0.15 Hz) and high-frequency (HF; 0.15-0.4 Hz) bands was calculated. All these variables were normalized by sex and age.

**Figure 1:**
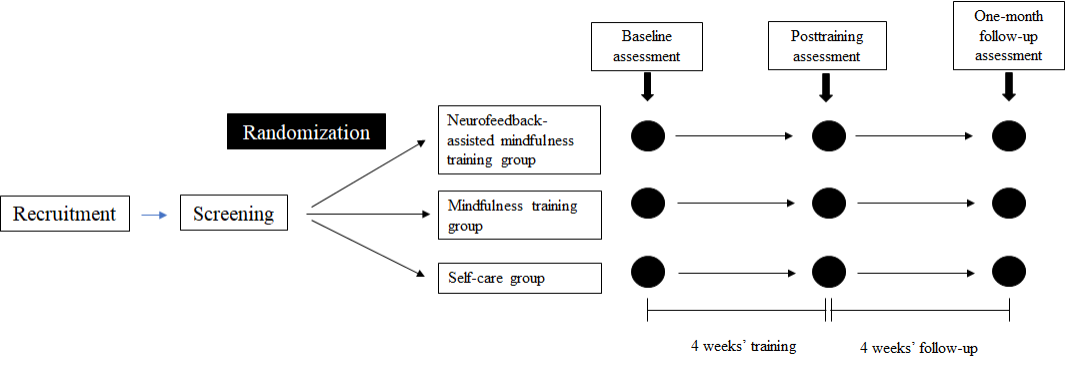
Assessment process of the study.

Throughout the study period, all self-reported psychological and physiological indices were measured at 3 time points: during enrollment (baseline assessment), immediately after training (posttraining assessment), and 4 weeks after training (1-month follow-up assessment; Figure 1). All outcome measures were obtained when participants visited the Seoul National University Hospital and the Seoul National University Bundang Hospital.

### Randomization and Intervention Conditions

Participants were assigned to 3 groups by 1:1:1 block randomization (stratified by organization and age) with randomly selected block sizes (3, 6, or 9) using REDCap (Research Electronic Data Capture; Vanderbilt University) tools hosted at the Medical Research Collaborating Center of Seoul National University Bundang Hospital. REDCap generates randomization codes with SAS (Statistical Analysis System; SAS Institute). The allocation sequence was concealed from the participants until they entered the trial, and from the investigators until the end of the study. Participants in the neurofeedback-assisted mindfulness training group (group 1) and mindfulness training-only group (group 2) were instructed to download a mobile app specifically developed for the research (developed by Omni C&S, Inc.). The app provides a mindfulness-based training program with an audio guide. In addition, for the neurofeedback-assisted mindfulness training group (group 1), headsets (OMNIFIT Brain; Omni C&S, Inc.) were provided. The neurofeedback function was embedded in the mobile app, with audio stimuli delivered to participants through the earphones of the headset. Group 1 participants received instructions on these processes during an educational session. Before initiating the training, the experimental groups (groups 1 and 2) received education to comprehend the concept of mindfulness and engaged in an hour-long practice session guided by a well-trained psychologist. All the participants in both groups attended 4 weekly meeting sessions with the psychologist, each lasting 30 minutes. In the meeting, the psychologist assessed the participants’ adherence to the training from the previous week and provided encouragement to those who reported lower levels of accomplishment. They also discussed any issues related to the training and the way forward. The control group participants, who practiced self-care (group 3), were given self-learning paper materials on stress management during their first visit, without any additional weekly meetings. These materials covered the definition of stress, signs, and symptoms of stress, as well as strategies for managing stress.

### Apparatus

EEG and HRV were measured using neuroNicle FX2 (Laxtha Inc.) [47]. The device is equipped with 2 EEG monopolar electrodes positioned over the left and right prefrontal areas (FP1 and FP2), along with a ground and reference electrode attached to a clamp on the right earlobe. Additionally, there is 1 pulse wave sensor (PPG) for measuring HRV, which is also attached to the same clamp. Both EEG and PPG data were recorded simultaneously. The raw EEG and PPG data were transmitted to the host device via Bluetooth technology (version 4.2) and subsequently analyzed to derive quantitative data, including power spectral components and neurofeedback indices. These data were then transmitted to the server of Omni C&S Inc., our research collaborating company, using Wi-Fi (wireless fidelity) technology. The device received medical device approval from the South Korean Ministry of Food and Drug Safety (authorization number 16-4837).

The OMNIFIT Brain is a headset device provided to participants in group 1 to aid in their neurofeedback training during the intervention. It is compatible with both Android (Google LLC/Alphabet Inc.) and iOS (Apple Inc.) operating systems. This headset features 2 EEG sensors and 1 PPG sensor, all operating at a sampling rate of 250 Hz, and it transmits neurophysiological data in real-time to the mobile app via Bluetooth technology. The app generates various neurofeedback indices, which are then relayed back to the headset, and the headset’s earphones provide auditory feedback to the participants.

### Mobile App

**Figure 2:**
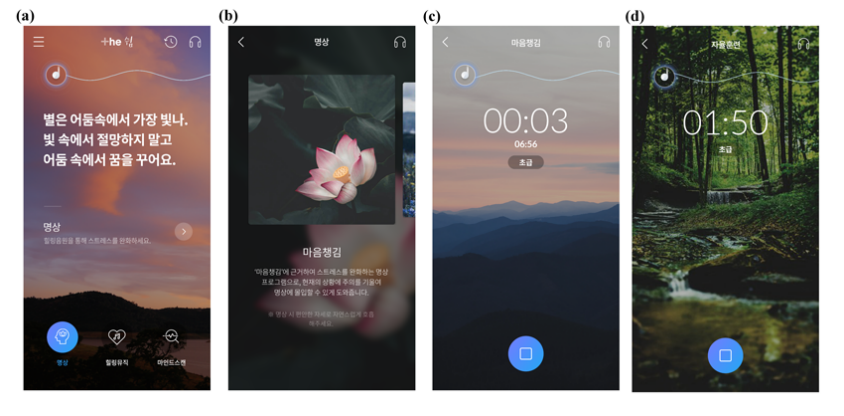
Images from the mobile app used in the mindfulness-based training program. (A) Main screen of the app; (B) introduction screen; (C) awareness program; and (D) self-exercise program.

The mindfulness-based training program comprised 3 components: awareness training (7 minutes), abdominal breathing (4 minutes), and body scan meditation (8 minutes), each accompanied by an audio guide (Figure 2). Following each session, participants had approximately 10 minutes for self-exercise. The order and frequency of these sessions were carefully structured as per the controlled research protocol, and participants were instructed to adhere to the provided schedule (refer to Table 1). The total duration of 1 training session, including self-exercise, was 20 minutes.

**Table 1:** Schedule of the 4-week mindfulness training program.

Programs			Frequency		Duration (days)		
**First week**							
	Breathing with self-exercise	Twice a day		3			
	Awareness with self-exercise	Twice a day		2			
	Body scan with self-exercise	Twice a day		2			
**Second week**							
	Breathing with self-exercise then awareness with self-exercise	Both, twice a day		7			
**Third week**							
	Breathing with self-exercise then body scan with self-exercise	Both, twice a day		7			
**Fourth week**							
	Self-exercise	Twice a day		7			

### Neurofeedback Function

For group 1, the neurofeedback function was facilitated using the OMNIFIT Brain during the self-exercise sessions. The neurofeedback training incorporated an alpha protocol designed to augment the power of alpha frequency compared with other frequency bands. The alpha wave is known to be associated with a state of relaxation [49], and alpha protocols are widely used in neurofeedback techniques aimed at reducing anxiety and stress, while enhancing mental performance [50]. Participants in group 1 were informed that they would receive a positive feedback sound (a 1-second ringing of a bell) when they were in a focused and relaxed state, whereas a negative feedback sound (a 1-second chirping sound of a cricket) would be provided when they were in a distracted and unrelaxed state.

During the self-exercise session, the ratio of alpha power (8-12 Hz) to high beta power (20-30 Hz) was calculated at 2-second intervals. Participants received a positive auditory neurofeedback sound through the earphones when the ratio of alpha power to high beta power reached or exceeded a level of 2.775. This criterion was validated in a prior clinical study involving 1500 Korean healthy volunteers, using the same EEG system, and the value was determined based on the distribution of t-scores within the population [51].

### Statistical Analysis

Demographic variables and baseline psychological measurements were analyzed using an ANOVA or Kruskal-Wallis test for continuous variables, while chi-square tests or Fisher exact tests were used to compare the 3 groups. Marital status, educational levels, and length of career were divided into 2 entities for simplicity. For all outcome measures, including psychological and physiological variables, a generalized estimating equation (GEE) was applied to examine the effects of interventions. A GEE is commonly used when the outcome variables are measured repeatedly from the same individual and probable dependencies are assumed between those variables [52]. In this study, we utilized GEE to examine the group, time, and their interaction term, as it allowed us to assess the differential group effect at each time point. In the absence of an interaction, we assessed the effect of the group. We applied a first-order autoregressive correlation structure and set the significance level at 2-tailed (paired) *P*<.05. All statistical analyses were conducted using IBM SPSS Statistics version 25 (IBM Corp.).

### Ethics Statement

The study protocol was approved by the Institutional Review Board of Seoul National University Hospital in Seoul and Seoul National University Bundang Hospital in Seongnam, South Korea (approval number: B-1807-483-303). All participants were provided with information about the study and signed informed consent forms before participation. This study is registered in the ClinicalTrials.gov (NCT03787407).

## Results

**Figure 3:**
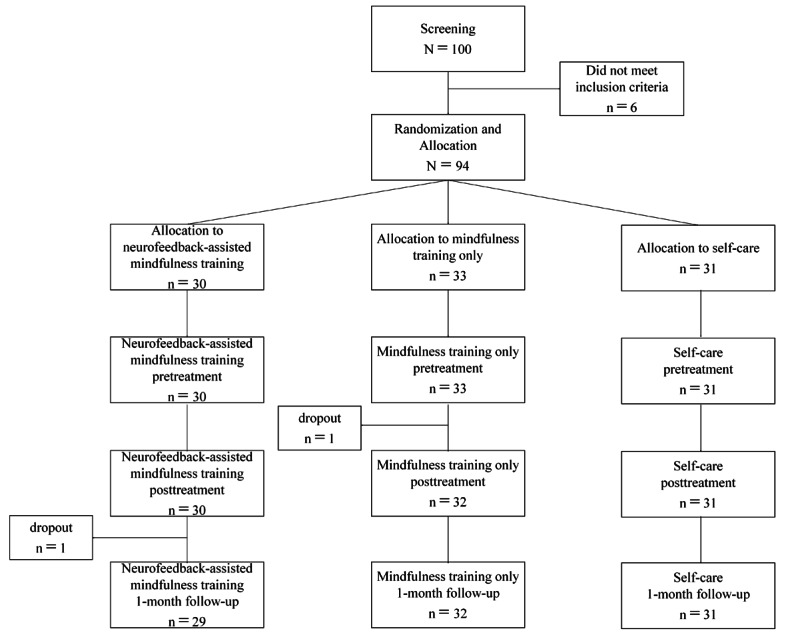
Flowchart of the study process. f/u: follow-up.

Of the 100 participants screened for this study, 6 did not meet the inclusion criteria. As a result, a total of 94 participants were initially included and randomly assigned to 1 of the 3 groups: 30 in group 1, 33 in group 2, and 31 in group 3. After the start of the intervention, 1 participant in group 2 dropped out due to compatibility issues between the mobile app and personal cellular phone. Thus, a total of 93 participants completed the training. One participant in group 1 dropped out before completing the 1-month follow-up assessment due to unemployment. Thus, 29 participants in group 1, 32 in group 2, and 31 in group 3 completed all assessments. Figure 3 presents a flow diagram of the study process. The dropout rates after training engagement were 3% (1/30), 3% (1/33), and 0% (0/31) for groups 1-3, respectively; this distribution was not statistically significant (*P*=.76) by Fisher exact test.

Demographic and clinical characteristics of the participants at baseline who completed all assessments are presented in Table 2. There were no significant differences in age (*P*=.76), sex (*P*=.67), marital status (*P*=.38), educational levels (*P*=.90), and length of career (*P*=.10) between the groups.

**Table 2:** Demographic and clinical characteristics of each group at baseline.

Characteristics	Neurofeedback-assisted mindfulness training (n=29)	Mindfulness-only training (n=32)	Self-care (n=31)	*F* or chi-square (*df*) statistic	*P* value
Age (years), mean (SD)	39.72 (10.01)	37.66 (10.59)	38.65 (11.88)	*F*=0.275 (2, 89)	.76
Sex (female), n (%)	27 (93.10)	29 (90.63)	27 (87.10)	*χ*^2^=0.796 (2)	.67
Marital status: unmarried, n (%)	11 (37.93)	18 (56.25)	14 (45.16)	*χ*^2^=1.913 (2)	.38
Education >college education, n (%)	27 (93.10)	29 (90.63)	27 (87.10)	*χ*^2^=0.203 (2)	.90
Length of career >3 years, n (%)	28 (96.55)	25 (78.13)	25 (80.65)	*χ*^2^=4.583 (2)	.10
Perceived Stress Scale score, mean (SD)	21.52 (4.38)	20.78 (4.82)	22.55 (4.95)	*F*=1.104 (2, 89)	.33
Brief Resilience Scale score, mean (SD)	19.72 (3.32)	19.97 (3.91)	19.81 (4.21)	*F*=0.033 (2, 89)	.96
Korean Version of the Mindfulness Attention Awareness Scale score, mean (SD)	65.28 (12.23)	60.72 (8.31)	57.81 (11.46)	*F*=3.664 (2, 89)	.03^a^
Korean Emotional Labor Scale score, mean (SD)	52.89 (7.20)	53.01 (11.89)	53.46 (15.09)	*F*=0.013 (2, 89)	.98
Korean Standard Occupational Stress Scale score, mean (SD)	53.33 (7.20)	51.69 (4.51)	48.90 (6.49)	*F*=4.016 (2, 89)	.02^b^
Athens Insomnia Scale score, mean (SD)	14.48 (3.12)	14.50 (2.23)	14.90 (3.35)	*F*=0.203 (2, 89)	.81
9-item Patient Health Questionnaire score, mean (SD)	5.41 (4.46)	6.34 (4.09)	6.77 (4.30)	*F*=0.761 (2, 89)	.47

^a^Post hoc test using the Tukey method was performed. The neurofeedback-assisted mindfulness training group exhibited higher scores on the Mindfulness Attention Awareness Scale compared with the self-care group.

^b^Post hoc test using the Tukey method was performed. The neurofeedback-assisted mindfulness training group exhibited higher scores on the Occupational Stress Scale compared with the mindfulness training group; the mindfulness training group exhibited higher scores compared with the self-care group.

**Figure 4:**
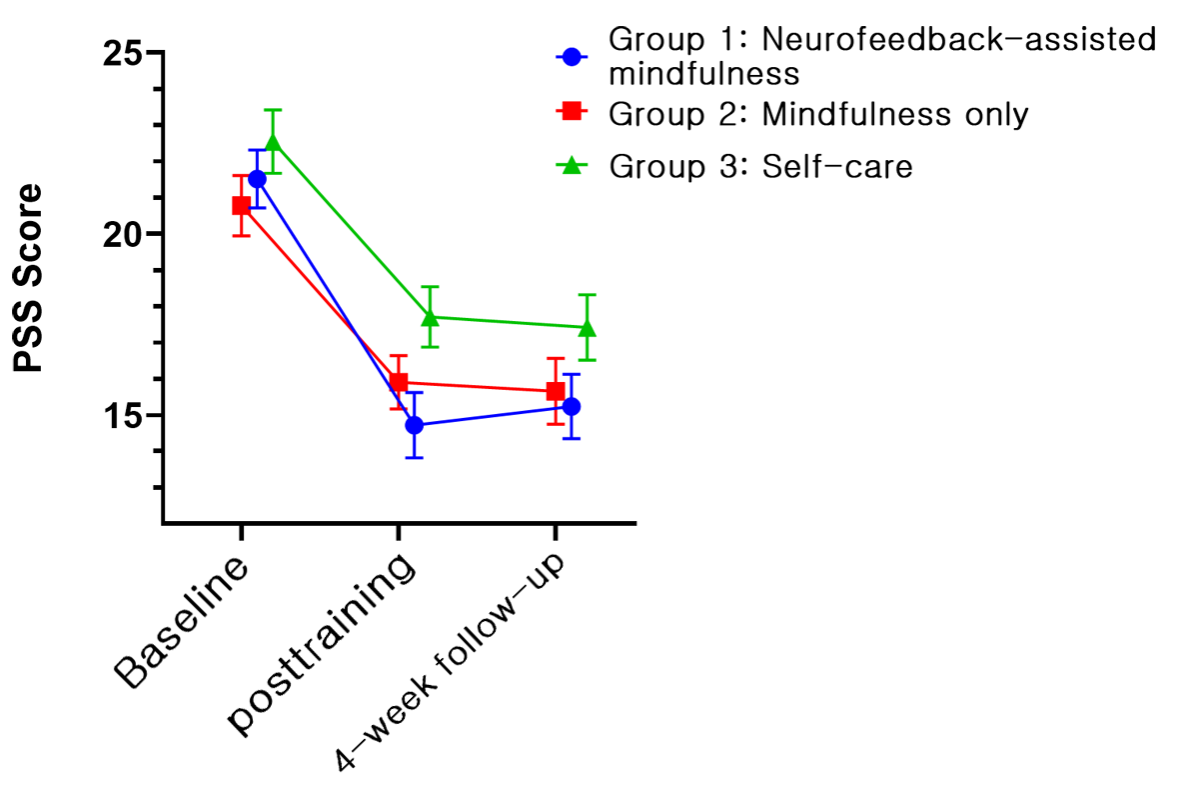
Perceived Stress Scale (PSS) scores across time according to condition (least squares means and SE). Group 1: neurofeedback-assisted mindfulness training; group 2: mindfulness training only; group 3: self-care.

**Figure 5:**
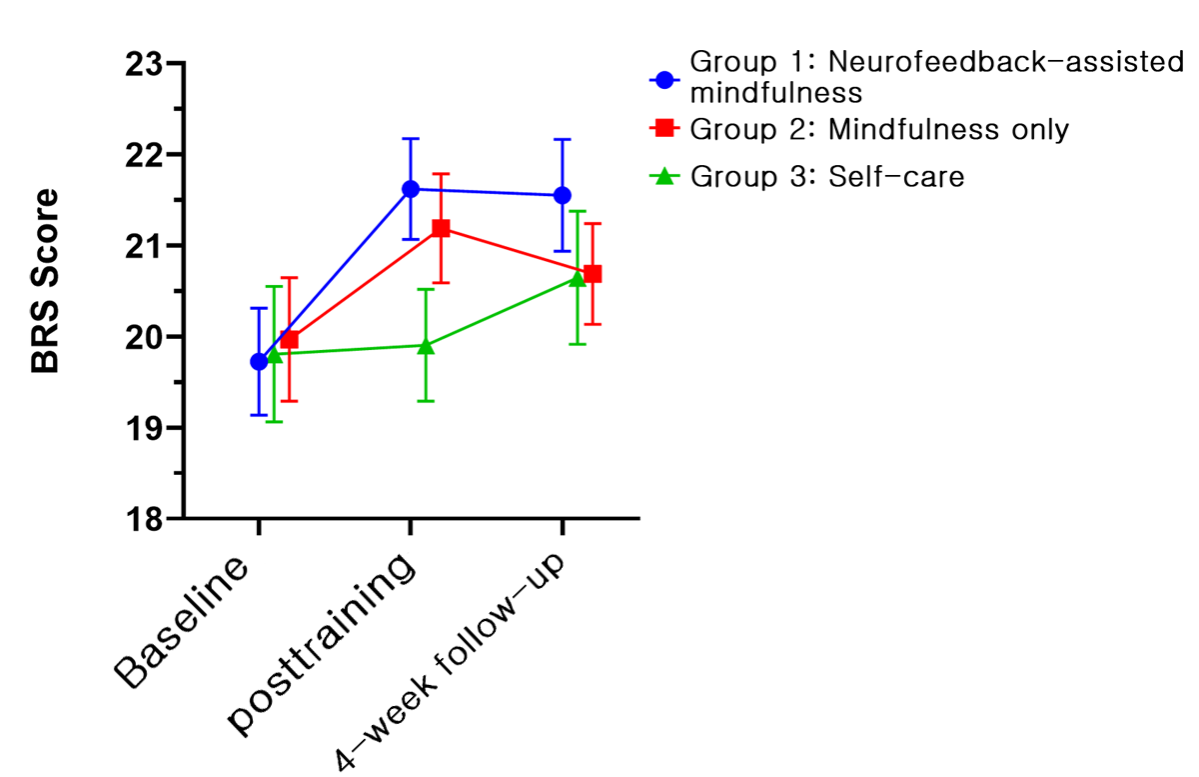
Brief Resilience Scale (BRS) scores across time according to condition (least squares means and SE). Group 1: neurofeedback-assisted mindfulness training; group 2: mindfulness training only; group 3: self-care.

**Figure 6:**
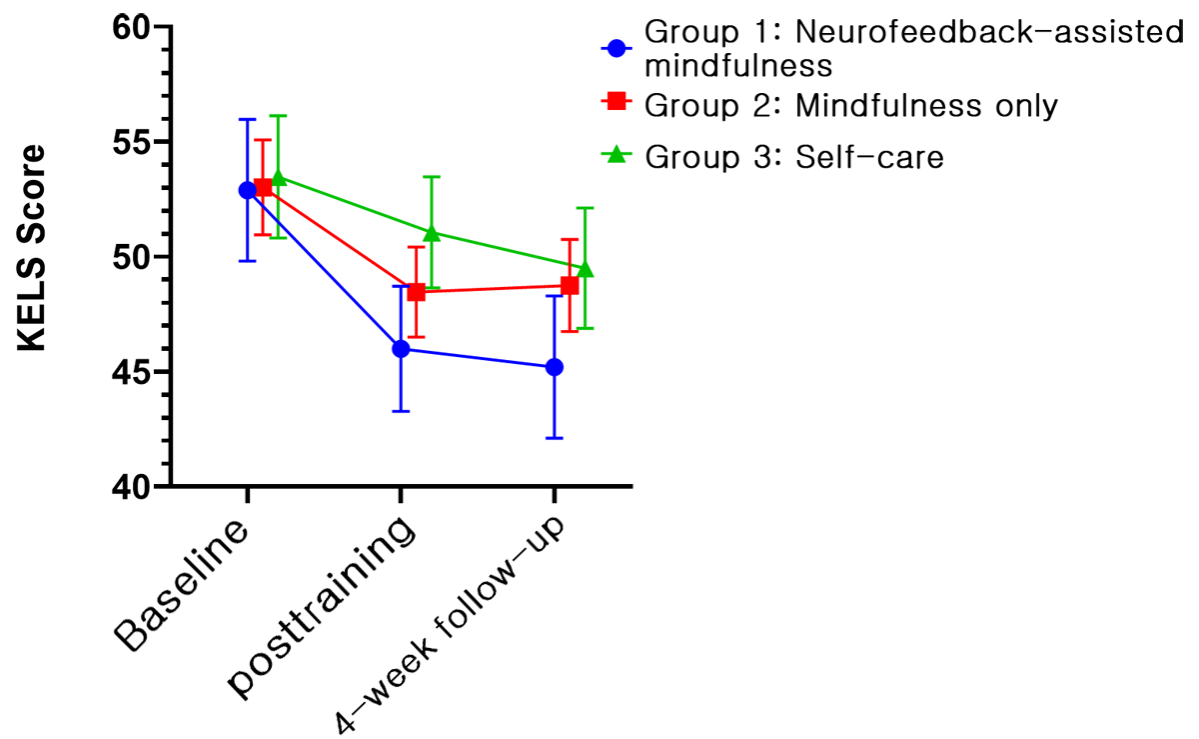
Korean Emotional Labor Scale (KELS) scores across time according to condition (least squares means and SE). Group 1: neurofeedback-assisted mindfulness training; group 2: mindfulness training only; group 3: self-care.

**Figure 7:**
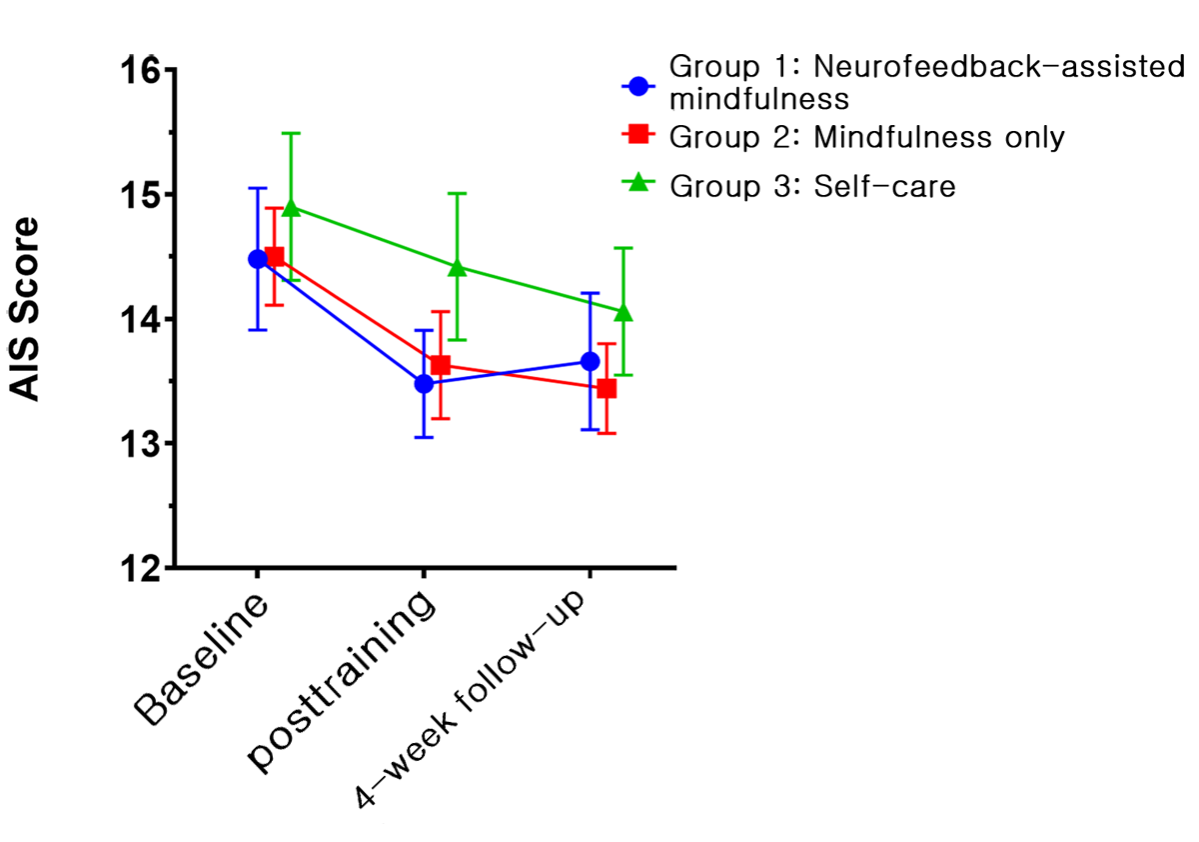
Athens Insomnia Scale (AIS) scores across time according to condition (least squares means and SE). Group 1: neurofeedback-assisted mindfulness training; group 2: mindfulness training only; group 3: self-care.

**Figure 8:**
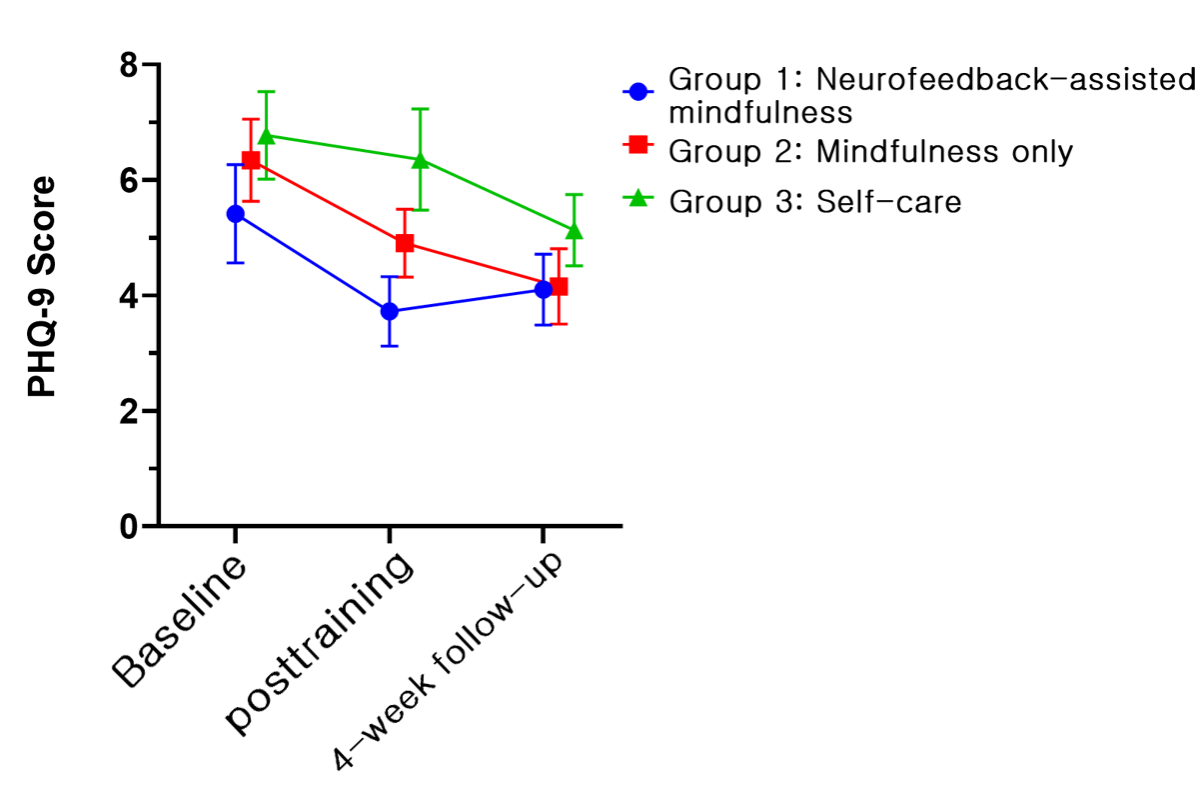
9-item Patient Health Questionnaire (PHQ-9) scores across time according to condition (least squares means and SE). Group 1: neurofeedback-assisted mindfulness training; group 2: mindfulness training only; group 3: self-care.

In all groups, 5 psychological variables, namely, PSS, BRS, KELS, AIS, and PHQ-9, showed improvements. Figures 4-8 depict the changes in PSS, BRS, KELS, AIS, and PHQ-9 scores across time. In the GEE model with interaction terms, neither the interaction (time × group) nor a main effect of group difference was found in the PSS score. Only the main effect of time was statistically significant in PSS (Wald *χ*_2_^2^=40.63, *P*<.001). All 3 groups showed a lower PSS score after the intervention (Wald *χ*_2_^2^=107.167, *P*<.001). For BRS (Figure 5), a significant interaction was observed (time × group, Wald *χ*_4_^2^=10.846, *P*=.02). In the post hoc analysis, a significant difference was found between group 1 (least squares mean [LSM] 21.62, SE 0.55) and group 3 (LSM 19.90, SE 0.61) at the posttraining assessment (*P*=.008). In addition, a significant effect of time (Wald *χ*_2_^2^=31.238, *P*<.001) was found, indicating that the resilience scores increased for both groups 1 and 2. Moreover, BRS scores at posttraining (LSM 21.62, SE 0.55) and 1-month follow-up (LSM 21.55, SE 0.61) assessments in group 1 revealed significant differences compared with the baseline assessment (LSM 19.72, SE 0.59, *P*<.001). In group 2, the score of the posttraining assessment was the highest (LSM 21.19, SE 0.60) and differed significantly from the baseline assessment (LSM 19.97, SE 0.68, *P*=.01), but not at the 1-month follow-up assessment (LSM 20.69, SE 0.55, *P*=.18). By contrast, we found no interaction in KELS, AIS, and PHQ-9 scores. However, the main effects of time were statistically significant in KELS (Wald *χ*_2_^2^=17.061, *P*<.001; Figure 6), AIS (Wald *χ*_2_^2^=14.391, *P*<.001; Figure 7), and PHQ-9 (Wald *χ*_2_^2^=16.395, *P*<.001; Figure 8) scores. No improvements were found in K-MAAS and KOSS scores. These results are summarized in Table 3.

**Table 3:** Generalized estimating equation model results of psychological variables.

Variables	Effects	Wald chi-square (*df*) statistic	*P* value
**PSS^a,b^**			
	Group × time interaction	2.472 (4)	.64
	Group	4.293 (2)	.11
	Time	107.167 (2)	<.001
**BRS^c,d^**			
	Group × time interaction	10.846 (4)	.02
	Group	0.075 (2)	.96
	Time	31.238 (2)	<.001
**KELS^e^**			
	Group × time interaction	3.491 (4)	.47
	Group	0.591 (2)	.74
	Time	17.061 (2)	<.001
**AIS^a,f^**			
	Group × time interaction	1.947 (4)	.74
	Group	0.875 (2)	.64
	Time	14.391 (2)	<.001
**PHQ-9^a,g^**			
	Group × time interaction	8.555 (4)	.07
	Group	2.679 (2)	.26
	Time	16.395 (2)	<.001
**K-MAAS^a,h^**			
	Group × time interaction	4.640 (4)	.32
	Group	9.139 (2)	.01
	Time	2.861 (2)	.23
**KOSS^a,i^**			
	Group × time interaction	5.055 (4)	.28
	Group	4.817 (2)	.08
	Time	3.067 (2)	.21

^a^Results of group and time effects were derived from the model without interaction.

^b^PSS: Perceived Stress Scale.

^c^Results of group and time effects were derived from the model with interaction.

^d^BRS: Brief Resilience Scale.

^e^KELS: Korean Emotional Labor Scale.

^f^AIS: Athens Insomnia Scale.

^g^PHQ-9: 9-item Patient Health Questionnaire.

^h^K-MAAS: Korean version of the Mindfulness Attention Awareness Scale.

^i^KOSS: Korean Standard Occupational Stress Scale.

**Figure 9:**
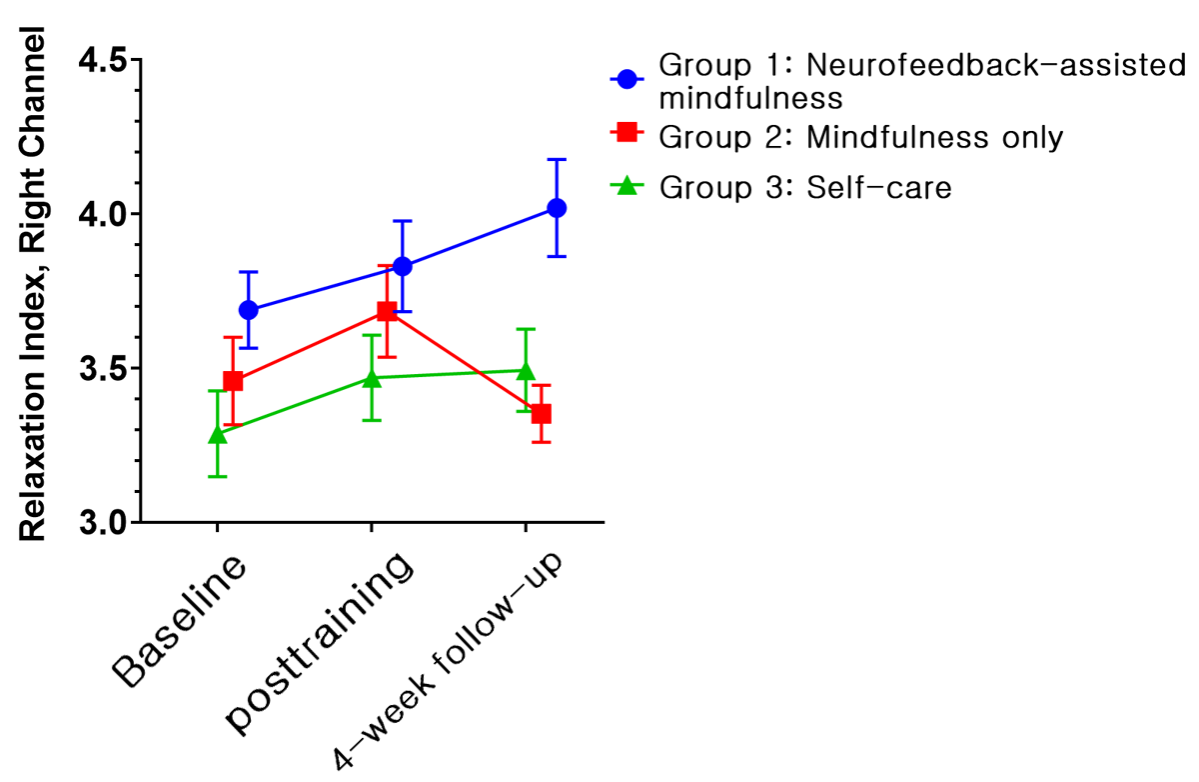
Relaxation Index across time according to condition (least squares means and SE). Group 1: neurofeedback-assisted mindfulness training; group 2: mindfulness training only; group 3: self-care.

**Figure 10:**
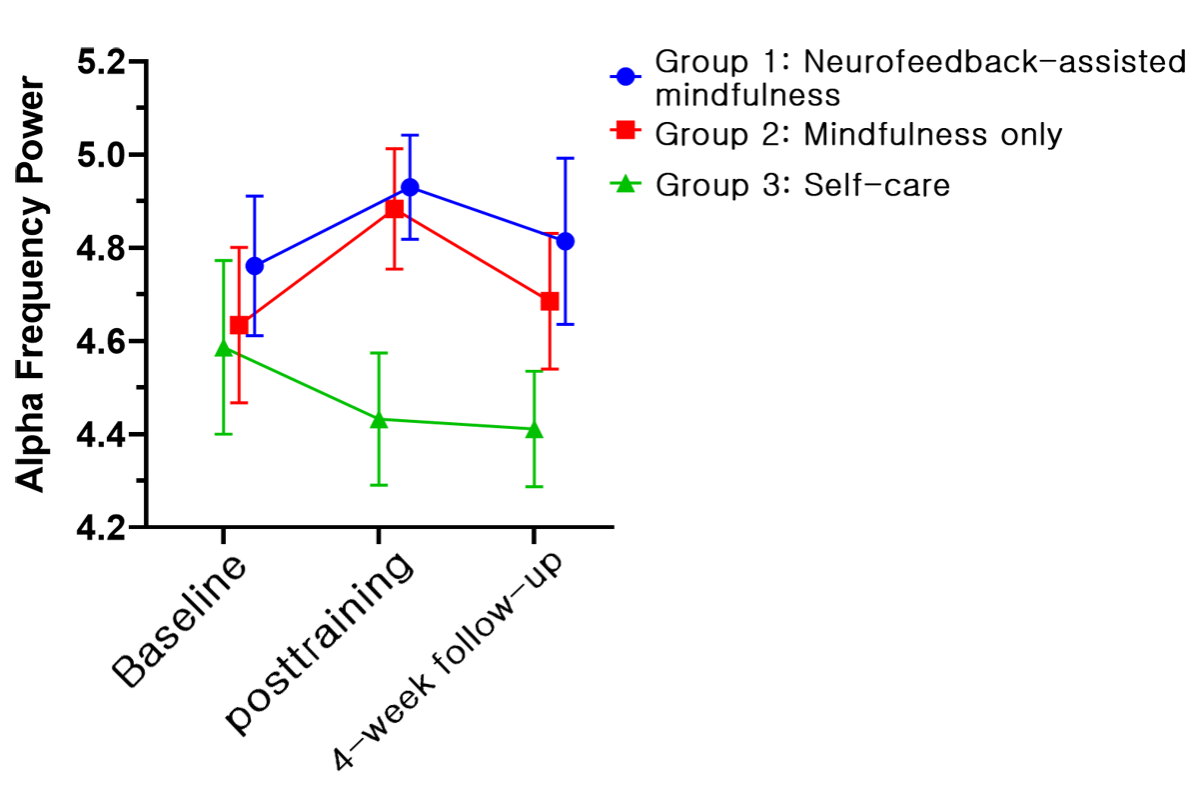
Power of the alpha frequency band of the right electroencephalogram channel (least squares means and SE). Group 1: neurofeedback-assisted mindfulness training; group 2: mindfulness training only; group 3: self-care.

**Figure 11:**
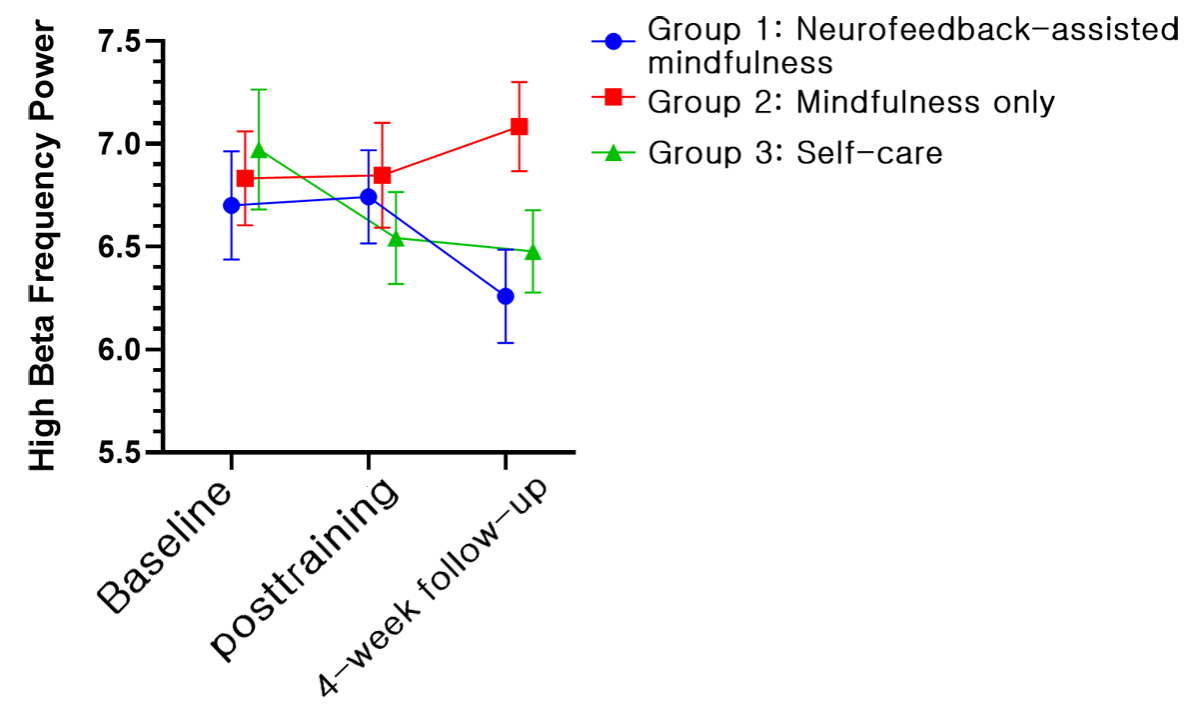
Power of the high beta frequency band of the right electroencephalogram channel (least squares means and SE). Group 1: neurofeedback-assisted mindfulness training; group 2: mindfulness training only; group 3: self-care.

**Figure 12:**
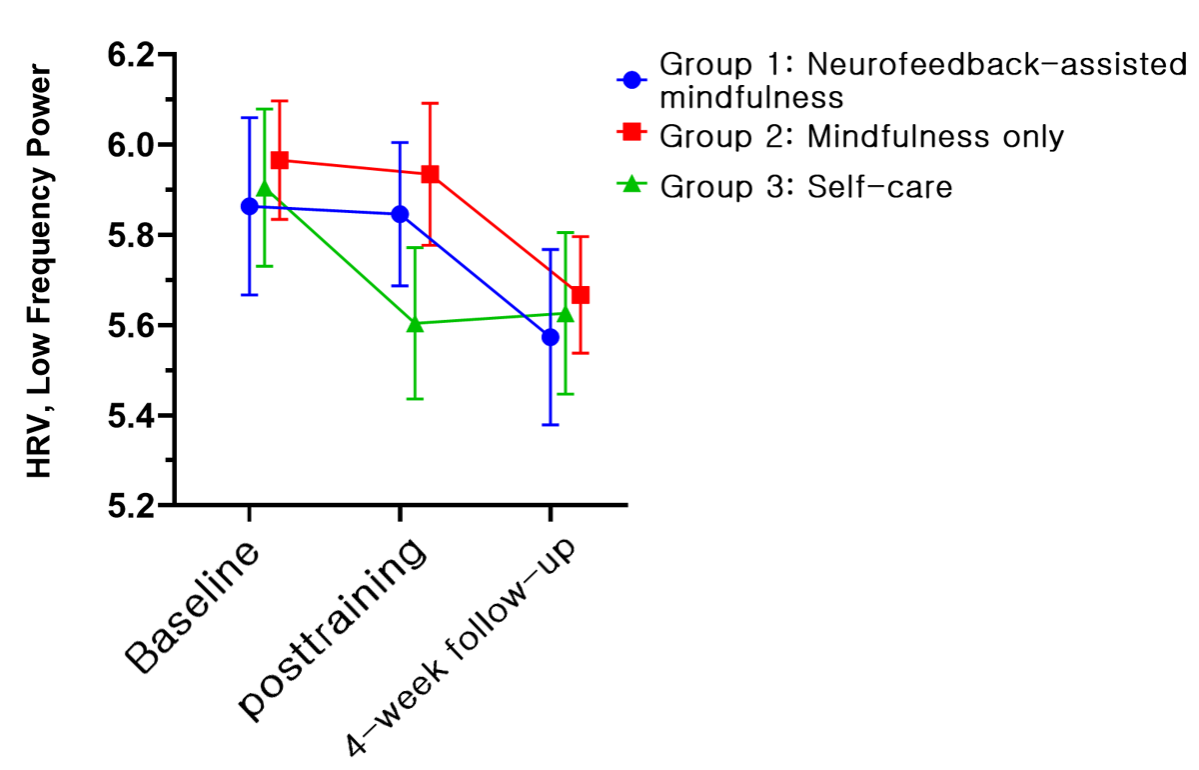
Low frequency power of heart rate variability across time according to condition (least squares means and SE). Group 1: neurofeedback-assisted mindfulness training; group 2: mindfulness training only; group 3: self-care.

The EEG and HRV data for 25 participants were excluded from the final analysis; one of the 3 measurements (ie, baseline assessment, posttraining assessment, and 1-month follow-up assessment) was not available due to either inappropriate length or continuity of data after removal of artifacts for analysis. Thus, the physiologic data for 67 participants (22 in group 1, 22 in group 2, and 23 in group 3) were obtained. However, no significant results were obtained in terms of time × group interactions when both EEG and HRV data were subjected to GEE analysis in the model. Conversely, irrespective of interactions, the relaxation index of the right prefrontal channel showed significant effects by group (Wald *χ*_2_^2^=9.235, *P*=.009) and time (Wald *χ*_2_^2^=6.255, *P*=.04; Figure 9). In the post hoc analysis, group 1 (LSM 3.87, SE 0.11) showed a significant difference compared with groups 2 (LSM 3.47, SE 0.10, *P*=.009) and 3 (LSM 3.42, SE 0.12, *P*=.007). In terms of time, a significant difference (*P*=.017) was found between the baseline (LSM 3.48, SE 0.08) and posttraining (LSM 3.66, SE 0.08, *P*=.01) values. In an additional analysis, we examined differences between groups with relative changes compared with baseline. A significant time × group interaction (Wald *χ*_2_^2^=6.947, *P*=.03) was found. In the post hoc analysis, the relaxation index changed significantly (*P*=.03) at both posttraining (LSM 0.24, SE 0.12, *P*=.04) and 1-month follow-up assessments (LSM 0.43, SE 0.15 *P*=.003) compared with the baseline measurement only in group 1. Furthermore, we observed a significant group difference (Wald *χ*_2_^2^=9.609, *P*=.008) at the 1-month follow-up assessment. Group 1 demonstrated the highest increase in the index (LSM 0.43, SE 0.15), in contrast to group 2 (LSM –0.11, SE 0.10) and group 3 (LSM 0.12, SE 0.10). We illustrated the changes of alpha and high beta frequency power, which are components of the relaxation index, along with the serial measurements. Between baseline and the posttraining assessment, the alpha power of groups 1 and 2 increased, whereas it decreased in group 3 (Figure 10). In the high beta frequency band, the power of groups 1 and 2 remained similar, but that of group 3 decreased (Figure 11). In the next period, between the posttraining and 1-month follow-up assessments, the alpha frequency band of groups 1 and 2 moved downward together (Figure 10), but in the beta frequency band, they moved separately in opposite directions (Figure 11). LF of HRV parameters showed a significant effect by time (Wald *χ*_2_^2^=14.073, *P*=.001) in the model without an interaction term (Figure 12). However, no other significant results were revealed via the HRV parameters.

## Discussion

### Principal Findings

In this study, we found that a 4-week mindfulness-based, self-training program delivered via a mobile app effectively reduced stress and improved other psychological indices among employees. Additionally, we hypothesized that the neurofeedback-assisted training might enhance the effects of mindfulness training. A significant interaction (times × group) was found in the BRS between the neurofeedback-assisted mindfulness training group and the control group, with the former showing a significant improvement at the posttraining assessment, with continued improvements observed during the 1-month follow-up assessment period. Group 2 (which received mindfulness training only) also showed significant improvement at the posttraining assessment, but at the 1-month follow-up, the improvement was not significant. In the relaxation index, the neurofeedback-assisted mindfulness training group showed ongoing improvements compared with the baseline assessment, with the improvement having significant differences compared with other groups at the 1-month follow-up assessment.

### Comparison With Prior Studies

Originally, high-fidelity MBSR [53] required an 8-week training program with a 7.5-hour silent retreat during week 6. However, this type of program is difficult for general employees to participate in. To address this barrier, various mindfulness-based training programs have been developed, with shorter duration or incorporation of other kinds of programs. These modified programs have been proven to be effective [13]. In our study, the mobile app delivered 3 training modules (awareness training, abdominal breathing, and body scan) over 4 weeks. One of the primary outcome measures was resilience using BRS. Group 1 showed the largest improvement compared with other groups (Figure 5) at the second assessment. The group also showed a significant training effect compared with group 3, which was also the case at the 1-month follow-up assessment.

Previous studies have suggested that mindfulness training could enhance resilience [54-56]. In addition, one study has reported that increased mindfulness may be related to increased resilience and decreased burnout [54]. Another study has demonstrated that high resilience and higher mindfulness may be significant predictors of lower levels of burnout and psychological distress [57]. Moreover, resilience has been known to be correlated with less stress, better mental health, and more mindfulness [58].

The other outcome measures of our study were evaluated based on the effects of mindfulness intervention programs reported in previously published studies. Several studies have reported improvements in PSS scores after a 4-week mindfulness training program [59,60]. Regarding emotional labor, mindfulness-based intervention appeared to cause less emotional exhaustion [61,62]. One study reported the mediating role of emotional labor in the negative correlation between mindfulness and burnout [63]. Regarding the effect of mindfulness on sleep, while some evidence suggests improved sleep, a systematic review concluded that controlled studies have not consistently demonstrated positive effects of MBSR on sleep quality and duration [64]. There were no statistically significant differences between groups 2 and 3 in these psychological measures. This could be attributed to the self-learning effect observed in the control group, which received the self-learning materials. According to a recent qualitative review of 67 published studies, only 5 had an active control group (7%) [13]. There was only 1 study with a 3-arm design that included an inactive control group and an active control group. The design and total number of training hours in that study were similar to those of our study; however, it did not find significant effects of the mindfulness-based program, even in the experimental group [65]. Two other 2-armed studies with experimental and active control groups (education only) conducted over 8 weeks yielded mixed results. In one of these studies, no significant differences were found between the groups on the PSS [66], whereas in the other study, significant differences were observed between the 2 groups on work-related stress scales [67].

On the other hand, for the relaxation index of the right prefrontal channel, the neurofeedback-assisted mindfulness training group exhibited significant differences compared with the other groups at the 1-month follow-up assessment. We depict the changes in alpha and high beta frequency power in Figures 10 and 11, respectively. The alpha power of groups 1 and 2 moved in the same direction during the measurements, but the high beta power of groups 1 and 2 diverged after the posttraining assessment. Notably, the alpha and high beta power of the control group followed a completely different trajectory compared with groups 1 and 2. This transition resulted in a significant interaction, further emphasizing the differences between the groups. Group 1 demonstrated the highest relaxation index at the last assessment. In summary, our findings suggest that a 4-week mindfulness-based training program may increase the power of alpha frequency in groups 1 and 2. Several studies have indicated that mindfulness training may be associated with enhanced alpha and theta power, although changes in beta, delta, and gamma power may exhibit inconsistent patterns [28].

For the power of LF in HRV, all 3 groups exhibited a decreased pattern without significant differences between the groups or interactions (Figure 12). This suggests that neurofeedback-assisted mindfulness training did not demonstrate additional effects on HRV compared with the other groups. The power of the LF band in HRV is known to be influenced by parasympathetic and sympathetic nervous activity, as well as baroreflex function [68]. Interpretation of HRV should be approached with caution [69] due to various contextual factors, including artifacts [70], measurement techniques [71], and others. Several studies have reported decreased LF band power following mindfulness-based training programs [72,73], which aligns with our results. However, another study reported no significant changes in LF but observed an increase in the LF-to-HF ratio [74], which is not consistent with our findings.

### Suggestions and Limitations

In this study, we found that the effects of neurofeedback-assisted mindfulness training persisted even during the follow-up period. Future research examining these effects over a more extended duration would be valuable. Additionally, we only recruited full-time employees, which could be considered a limitation. Therefore, future studies would benefit from including a broader range of participants across different conditions, encompassing a more diverse array of physiological variables, and employing larger sample sizes.

### Conclusions

In this study, we developed a mindfulness training program embedded in a mobile app and verified its effectiveness over 4 weeks. The program delivered by the mobile app was effective on PSS, BRS, KELS, AIS, and PHQ-9 scores. The neurofeedback-assisted mindfulness training group showed a significant difference in BRS compared with the control group. In addition, the effect of neurofeedback-assisted mindfulness training on relaxation remained significant at the 1-month follow-up assessment. Therefore, we could suggest that a 4-week program via a mobile app may contribute to stress reduction of employees and could improve resilience and relaxation when the mindfulness training is supported by the neurofeedback function.

## Appendix

Multimedia Appendix 1 CONSORT (Consolidated Standards of Reporting Trials) checklist.

## Abbreviations

AIS: Athens Insomnia Scale
BRS: Brief Resilience Scale
EEG: electroencephalography
GEE: generalized estimating equation
HF: high frequency
HRV: heart rate variability
KELS: Korean Emotional Labor Scale
K-MAAS: Korean version of the Mindfulness Attention Awareness Scale
KOSS: Korean Standard Occupational Stress Scale
LF: low frequency
LSM: least squares mean
MBSR: mindfulness-based stress reduction
MINI: Mini International Neuropsychiatric Interview
PHQ-9: 9-item Patient Health Questionnaire
PPG: plethysmograph
PSS: Perceived Stress Scale
REDCap: Research Electronic Data Capture
SAS: Statistical Analysis System
Wi-Fi: wireless fidelity
